# *Leptospira interrogans* Binds to Cadherins

**DOI:** 10.1371/journal.pntd.0002672

**Published:** 2014-01-30

**Authors:** Karen Evangelista, Ricardo Franco, Andrew Schwab, Jenifer Coburn

**Affiliations:** 1 Graduate Program in Microbiology, Immunology, and Molecular Genetics, Medical College of Wisconsin, Milwaukee, Wisconsin, United States of America; 2 Division of Infectious Diseases, Department of Medicine, Medical College of Wisconsin, Milwaukee, Wisconsin, United States of America; University of Washington, United States of America

## Abstract

Leptospirosis, caused by pathogenic species of *Leptospira*, is the most widespread zoonosis and has emerged as a major public health problem worldwide. The adhesion of pathogenic *Leptospira* to host cells, and to extracellular matrix (ECM) components, is likely to be necessary for the ability of leptospires to penetrate, disseminate and persist in mammalian host tissues. Previous work demonstrated that pathogenic *L. interrogans* binds to host cells more efficiently than to ECM. Using two independent screening methods, mass spectrometry and protein arrays, members of the cadherin family were identified as potential *L. interrogans* receptors on mammalian host surfaces. We focused our investigation on vascular endothelial (VE)-cadherin, which is widely expressed on endothelia and is primarily responsible for endothelial cell-cell adhesion. Monolayers of EA.hy926 and HMEC-1 endothelial cells produce VE-cadherin, bind *L. interrogans in vitro*, and are disrupted upon incubation with the bacteria, which may reflect the endothelial damage seen *in vivo*. Dose-dependent and saturable binding of *L. interrogans* to the purified VE-cadherin receptor was demonstrated and pretreatment of purified receptor or endothelial cells with function-blocking antibody against VE-cadherin significantly inhibited bacterial attachment. The contribution of VE-cadherin to leptospiral adherence to host endothelial cell surfaces is biologically significant because VE-cadherin plays an important role in maintaining the barrier properties of the vasculature. Attachment of *L. interrogans* to the vasculature *via* VE-cadherin may result in vascular damage, facilitating the escape of the pathogen from the bloodstream into different tissues during disseminated infection, and may contribute to the hemorrhagic manifestations of leptospirosis. This work is first to describe a mammalian cell surface protein as a receptor for *L. interrogans*.

## Author Summary

Leptospirosis is a globally widespread bacterial infection caused by pathogenic species of the genus *Leptospira*. The disease manifestations of leptospirosis range from mild, non-specific illness to a severe disease that includes multi-organ failure, widespread damage to blood vessels, and hemorrhage. Attachment to human or animal cells is likely to be important to the ability of the bacteria to spread and to cause disease. In this study, members of the cadherin family were identified as mammalian cell receptors that bind *Leptospira*. Cadherins are cell surface proteins that function to maintain cell-cell integrity by anchoring neighboring cells together. Disease-causing *L. interrogans*, but not the non-infectious *L. biflexa*, binds to cells that line blood vessels and VE-cadherin, the predominant cadherin found in this cell type. The binding of bacteria was reduced in the presence of antibodies against VE-cadherin, supporting the role of this protein in bacterial attachment. The attachment of *L. interrogans* to the inner lining of the vessels *via* VE-cadherin may result in damage, facilitating the escape of the pathogen from the bloodstream into different tissues, and may contribute to the hemorrhagic manifestations of leptospirosis. This work is first to identify a mammalian cell surface protein as a receptor for *L. interrogans*.

## Introduction

Leptospirosis is a globally widespread zoonotic infection caused by spirochetes of the genus *Leptospira*. The genus contains free-living non-pathogenic species as well as several species that are pathogenic for humans and/or animals. The disease in humans varies from a mild, non-specific, self-limited illness to an acute life-threatening infection (Weil's disease) with kidney failure, myocarditis, liver dysfunction, and sometimes pulmonary hemorrhage. Both domesticated and wild animals can serve as reservoirs from which humans acquire the disease, either through direct contact with the animals' tissue or fluids, or indirectly through contact with water or mud containing leptospires shed by reservoir animals in the urine. In addition to the costs to human life and health, leptospirosis can also lead to livestock and companion animal losses, potentially leading to adverse impacts on human well-being on several levels.

Persistently infected reservoir animals harbor the spirochete in the proximal convoluted tubules of the kidney and chronically excrete *Leptospira* through the urine. *Leptospira* species enter the body through mucous membranes of the eyes, nose or throat and *via* cuts or abrasions in the skin. During clinical disease, widespread damage to the endothelium may be seen (reviewed in [1]). It is likely that *Leptospira* interactions with endothelial and kidney proximal tubule epithelial cells are critical to the dissemination and persistence of the organism, but the mechanisms of these interactions remain poorly understood. The adhesion of *Leptospira interrogans* to endothelial, fibroblast, kidney epithelial, and monocyte-macrophage cell lines cultured *in vitro* has been demonstrated [2]–[8]. In most cases, virulent strains bind more efficiently than avirulent or non-pathogenic (saprophytic) strains [2], [8]–[11]. The attachment of saprophytic strains such *L. biflexa* sv. Patoc to host cell monolayers is considered by many to be non-specific, as in some systems the bacteria bind inert surfaces like glass and plastic just as efficiently [2], [10]. Even if cell specific binding by *L. biflexa* is seen, it is less efficient than that by *L. interrogans*. In addition, penetration of MDCK and endothelial cell layers was also associated with infectious vs. saprophytic *Leptospira* [11], [12]. The majority of studies on *Leptospira* adherence, however, have focused on host proteins found in the plasma or extracellular matrix (ECM) [3], [13]–[38], not the cell surface receptors that may allow the bacteria to alter mammalian signaling cascades to their own benefit. In this work, we focused on identification of mammalian cell surface proteins that serve as receptors for *Leptospira interrogans*.

## Materials and Methods

### Reagents

The purified proteins tested in this study were superfibronectin and fibronectin from human plasma (Sigma-Aldrich, St. Louis, MO); human recombinant vitronectin (Upstate, Lake Placid, NY); recombinant VE-cadherin (Human CD-144, cadherin-5), recombinant E-cadherin (Human cadherin-1), and recombinant ICAM-2 (Human CD102) (Bioclone Inc, San Diego, CA); Human integrins α_3_β_1_, α_5_β_1_, α_v_β_3_ and α_v_β_5_ (Chemicon International, Temecula, CA), and α_IIb_β_3_ (purified in the laboratory as previously described [39]). We observed that there is some batch-to-batch variation in the different commercial preparation in *L. interrogans* binding, as was the case for *B. burgdorferi* binding to integrins (unpublished observations). Cell culture reagents were purchased from Invitrogen/Life Technologies (Grand Island, NY), other reagents were purchased from Sigma-Aldrich or Thermo Fisher Scientific (Waltham, MA).

### Bacterial culture

*Leptospira interrogans* serovar Copenhageni (pathogenic, strain Fiocruz L1–130) was provided by Dr. David Haake (UCLA, Los Angeles, CA). This strain was reisolated by infection of hamsters, and then stored at passage 1 and 2 in liquid nitrogen. Frozen aliquots were thawed and passaged in liquid Ellinghausen-McCullough-Johnson-Harris (EMJH) medium [40] supplemented with rabbit serum and 5-fluorouracil. The bacteria used for the radioactive binding assays were at low passage (≤6 passages from hamster isolates). This strain has a 50% lethal dose range of 37–10^4^ in hamsters [12], [41], [42] and the genome sequence was previously reported [43]. *L. interrogans* serovar Canicola (strain 23606, known to be virulent according to the American Type Culture Collection (ATCC) and *L. biflexa* serovar Patoc (avirulent; strain 23582) were obtained from the ATCC (Manassas, VA).

Radiolabeled bacteria were prepared by supplementing the medium with ^35^S methionine plus cysteine (PerkinElmer, Boston, MA) and stored in aliquots at −80°C as previously described [9]. For individual experiments, aliquots of bacteria were thawed, resuspended in 10 ml of EMJH medium and pelleted for 30 minutes at 2,683× g. The supernatant was removed and bacterial pellet was resuspended in Dulbecco's modified Eagle medium (DMEM) supplemented with bovine serum albumin (BSA) to 1%. Motile leptospires were counted by dark-field microscopy using a Petroff-Hausser counting chamber. The bacterial suspension was adjusted to 7×10^6^/ml and dispensed 50 µl/well in 96-well plates. There is some batch-to-batch variation in the radiolabeling and binding efficiencies of ^35^S labeled leptospires, so data are shown as the % inoculum bound rather than absolute number of bacteria. All manipulations of living *Leptospira* were performed within a biosafety cabinet.

### Mammalian cell culture

The human macrovascular endothelial cell line EA.hy926, provided by Dr. C.-J. Edgell (University of North Carolina, Chapel Hill, NC) [44], was grown in DMEM with high glucose supplemented with 10% heat-inactivated fetal bovine serum (FBS) (Gibco, Grand Island, NY), 1 U/mL penicillin, 1 µg/mL streptomycin, 2 mM L-glutamine, and hypoxanthine-aminopterin-thymidine (HAT) medium supplement (Sigma-Aldrich) in a humidified atmosphere of 5% CO_2_ as previously described [11]. The human microvascular endothelial cell line HMEC-1 [45] was grown in MCDB 131 medium and supplemented with 15% heat-inactivated FBS (Hyclone, Logan, UT), 2 mM L-glutamine, 10 ng/ml epidermal growth factor (EGF), 1 µg/ml hydrocortisone and 25 mM HEPES. The human Caco2_BBE_ intestinal carcinoma cell line, a kind gift from Dr. Michael Dwinell (Medical College of Wisconsin), was cultured in DMEM (4 g/L glucose) supplemented with 10% (v/v) heat-inactivated FBS (Gibco, Grand Island, NY), 2 mM L-glutamine, and 10 mM HEPES (Gibco, Grand Island, NY). The human epithelial cell lines HEp-2 (laryngeal carcinoma) and HK-2 (proximal renal tubule) were purchased from the ATCC. HEp-2 cells were cultured in Eagle's Minimum Essential Medium supplemented with 10% FBS while HK-2 cells were grown in keratinocyte serum-free medium supplemented with 50 µg/ml bovine pituitary extract and 5 ng/ml human recombinant EGF (Gibco, Grand Island, NY). All cell lines were grown in the presence of 100 U/ml penicillin and 100 µg/ml streptomycin at 37°C under 5% CO_2_.

For bacterial adhesion assays, the cells were plated at 50% confluence in 96 well plates and then incubated for 2 days to reach confluence. The cells were incubated for an additional 1–2 days at 37°C, 5% CO_2_ to reach post-confluence, while viability was maintained. Prior to the addition of radiolabeled leptospires, the cell layers were washed 3× with 200 µl/well phosphate-buffered saline, and incubated for one hour in culture medium without antibiotics.

### Bacterial adhesion assays

Purified proteins were diluted to 0.01, 0.03 or 0.1 µM using sterile HBSC (25 mM HEPES pH 7.6, 150 mM NaCl, 1 mM MgCl_2_, 1 mM MnCl_2_, and 0.25 mM CaCl_2_) supplemented with 0.01 trypsin inhibitory units/mL (TIU/ml) aprotinin and 1 mM benzamidine. The proteins were plated on pre-chilled 96-well Linbro plates (MP Biomedicals, Solon, OH) at 50 µl/well in quadruplicate along with a HBSC buffer-only control and BSA (plated at 0.1 µM) controls. The plates were left overnight at 4°C, then washed with 200 µl/well HBSC and blocked with 1% BSA in DMEM for 1 hour at 4°C on a rocker. The wells were then washed with HBSC buffer before adding the bacteria. ^35^S-labeled leptospires were added at approximately 3.5×10^5^ bacteria/well in DMEM +1% BSA. In binding saturation assays, 1×10^3^–1×10^6^ radiolabeled *L. interrogans* were incubated with wells coated with 0.03 µM receptor. To quantify the inoculated bacteria, the inoculum suspension was also added to 8 wells on a 96-well Luma scintillation plate (Packard, Meriden, CT). The Luma plate was left to dry at room temperature. The assay plate was centrifuged at 670× g for 20 minutes and then incubated for 1 hour at 37°C under 5% CO_2_. Unbound bacteria were then washed away with 3×200 µl/well with HBSC. Sodium dodecyl sulfate (SDS) 1% in dH_2_O was added to each well for 10 minutes to solubilize the bound bacteria. The contents of each well were transferred to a corresponding well in a Luma plate and left to dry at room temperature. Bound bacteria were quantified by scintillation counting in a plate counter (Perkin Elmer Microbeta^2^ 2450 Plate Reader), and the percent inoculum bound determined for each well in a quadruplicate set by subtracting the no bacteria background, then dividing by the average of the inoculum well counts.

For inhibition assays, purified VE-cadherin immobilized on plates was blocked with 1% BSA in DMEM for 1 hr at room temperature. Function blocking anti-VE-cadherin antibody clone BV9 (Abcam, Cambridge, MA) at different concentrations was added to wells and incubated for 1 hour at 37°C, 5% CO_2_. 3.5×10^5^ radiolabeled *L. interrogans* resuspended in DMEM supplemented with 1% BSA was added to each well, and incubated for 1 hr at 37°C, 5% CO_2_. The bound bacteria were determined as described above.

Binding to cell layers was performed using essentially the same protocol covered earlier. The cell monolayers were infected with 3.5×10^5^ or 7×10^5^ radiolabeled *L. interrogans* serovar Copenhageni which resulted in a multiplicity of infection (MOI) of 10 or 20, as detailed in the Figure Legends. For competition assays, post-confluent cell monolayers cultured on 96-well plates were incubated for 30 minutes at 37°C, 5% CO_2_ with function blocking (anti-VE-cadherin clone BV9, Abcam, Cambridge, MA) or control (purified mouse IgG2a, Chemicon International, Temecula, CA) antibodies resuspended in phosphate-buffered saline (PBS). ^35^S *L. interrogans* resuspended in PBS instead of DMEM, was added to monolayers pretreated with antibodies at an MOI = 10. The integrity of the monolayers was assessed visually prior to the addition of SDS. Bacterial attachment was assessed as previously described.

### Endothelial cell membrane protein extraction

The human macrovascular endothelial cell line EA.hy926 (≤10 passages from the source) was grown to confluence in 10 BD Falcon T225 tissue culture flasks (Fisher Scientific, Hanover Park, IL) at 37°C under 5% CO_2_. The cells were washed with PBS and lifted from the tissue culture flasks with 10 mM EDTA in PBS. The cells were pelleted in 2 tubes and collected by centrifugation at 168× g for 10 minutes at ambient temperature. The supernatant was discarded and each pellet was resuspended in 20 mL of HBSC supplemented with 0.01 TIU/ml aprotinin and 1 mM benzamidine. The tubes were centrifuged again at 168× g for 10 minutes and the supernatant discarded. The pellets were each resuspended in 500 µL of HBSC with aprotinin, benzamidine, and PMSF (phenylmethane sulfonyl fluoride) to a final concentration of 1 mM and transferred to a 1.5 mL microfuge tube. The tubes were then placed in a beaker filled with 95% ethanol and dry ice. Once the contents had frozen, they were quickly thawed under running warm water, taking care to keep the contents cool by constant inversion. The freeze-thaw procedure was repeated once more, then the lysed cell samples were centrifuged at 16,100× g for 5 minutes at 4°C. The supernatant was completely removed, and the pellet was resuspended in 500 µL of 25 mM octylglucoside in HBSC (HBSCO) with aprotinin and benzamidine, then rocked gently for 1 hour at 4°C. The tubes were then centrifuged at 16,100× g for 5 minutes at 4°C and the supernatant containing the soluble proteins was transferred to 1.5 mL microfuge tubes and stored at −80°C. Aliquots of each fraction were tested for *L. interrogans* binding activity, and the octylglucoside supernatant, containing the majority of the activity (not shown), was further fractionated. Extraction with Triton X-100 abolished the ability to bind *L. interrogans* (data not shown).

### Protein fractionation and LC-MS analysis

A HiTrap DEAE FF 5 mL column (GE Healthcare, Piscataway, NJ) was connected to a GE AKTA Explorer 100 FPLC Protein Purification System with Unicorn 5.2 workstation software (GE Healthcare) at 4°C. The lines were washed with 5 mL of dH_2_O followed by 5 mL of HBSCO. A 500 µl sample of the EA.hy926 octylglucoside supernatant was then injected onto the column. HBSCO was run through the column until the UV absorbance decreased to a steady state, indicating that all the unbound protein had been washed off. A gradient formed by HBSCO alone and supplemented with 1 M NaCl was then allowed to run for 100 min.

Attachment of *L. interrogans* to the different endothelial membrane protein fractions was assessed by dilution of samples of the fractions to 1∶10 in HBSC. The diluted fractions were then plated 50 µl/well in pre-chilled Linbro plates and incubated overnight at 4°C. After washing and blocking as described earlier, wells were incubated with 3.5×10^5^ radiolabeled *L. interrogans* for 1 hr at 37°C, 5% CO_2_. The percentage of inoculated bacteria bound was determined as described above.

For LC-MS analysis of fractions to which *L. interrogans* bound, the fractions with activity were separated by SDS-PAGE [46] adjacent to neighboring fractions with no binding activity. The gels were stained with silver, and bands unique to the fractions with binding activity were excised and destained, then washed and treated with DTT followed by iodoacetamide. After washing and drying, the bands were digested with trypsin, and the resultant peptides were extracted, then analyzed by liquid chromatography-tandem mass spectrometry (LC-MS/MS) using a Surveyor NanoLC interfaced to an LTQ linear ion trap mass spectrometer (Thermo Scientific, Waltham, MA). Proteins were identified by searching the data against the human subset of the Uniprot 54 database using Sequest software (Thermo Scientific). Results of the database search were filtered with Epitomize then analyzed with Visualize, open-access software developed at the Medical College of Wisconsin. Proteins that met the following criteria were considered confident identifications: minimum peptide probability of 0.95, minimum protein probability of 0.95, and a minimum peptide count of 3.

### Protein array

Human protein arrays printed on glass slides were obtained from RayBiotech (Norcross, GA). A blocking solution of HBSC +1% BSA was added to each well for one hour. The solution was siphoned off using an ultrafine pipet tip, and then *L. interrrogans* serovars Copenhageni or Canicola at 10^10^ bacteria/mL in DMEM plus 1% BSA were added. The bacteria were incubated with the arrays at 37°C for one hour or three hours. The slides were gently rocked every half hour. At the end of the incubation, the bacteria were siphoned off gently and the wells were washed 4× with HBSC. Bound bacteria were fixed with 3% paraformaldehyde in PBS for 30 minutes. The wells were washed 4× with Tris-buffered saline (TBS; 25 mM Tris pH 7.5, 150 mM NaCl), then blocked with 1× TBS with 1% BSA. Rabbit anti-LipL32 (generous gift of Dr. David Haake) at 1∶10,000 dilution in TBS + BSA was incubated with the arrays for 1 hour on a rocker at 4°C. After washing the arrays with TBS, kit-supplied biotinylated anti-rabbit IgG was incubated for one hour at 4°C. The arrays were again washed with TBS, then probed with kit-supplied Alexa fluor 555-conjugated streptavidin for one hour at 4°C, per the RayBiotech instructions. After washing 4× with TBS, the slides were dried by centrifugation in the vertical position for three minutes at 1,000 rpm and sent to RayBiotech for scanning and analysis.

### Immunoblot analysis

The expression of receptors in various cell lines was determined by immunoblot analysis. Briefly, post-confluent cell monolayers were washed with PBS and collected with a cell scraper. The cells were homogenized in lysis buffer (50 mM HEPES, 150 mM NaCl, 1.5 mM MgCl_2_, 1 mM EGTA, 10% glycerol, 1% Triton X-100, 1 µg/ml leupeptin, 1 mM benzamidine and 0.01 TIU/ml aprotinin). Protein concentrations were determined using the Bradford Protein assay (Bio-Rad, Hercules, CA). Equal amounts of total cell lysate (15 µg total protein per lane) were resolved by 10% SDS-PAGE under reducing conditions. The proteins were transferred onto a polyvinylidene difluoride (PVDF) membrane (Immobilon, Millipore, Billerica, MA) and blocked with 5% milk in TBS overnight at 4°C. The membranes were probed with anti-pan cadherin antibody (polyclonal, dilution 1∶250; Invitrogen, Camarillo, CA), and antibodies against receptors VE-cadherin (clone BV9, dilution 1∶100; Novus, Littleton, CO), E-cadherin (clone HECD-1, dilution 1∶500; Calbiochem, San Diego, CA), N-cadherin (clone GC-4, dilution 1∶200; Sigma-Aldrich, St. Louis, MO) and ICAM-2 (polyclonal, dilution 1∶2,000; R&D Systems, Minneapolis, MN). Purified proteins were used to verify antibody reactivity by immunoblot when possible. As a loading control, the membranes were also probed with anti-GAPDH antibody (clone 14C10, dilution 1∶1,000; Cell Signaling Technology, Danvers, MA). Bound primary antibodies were detected with goat anti-rabbit, anti-rat or anti-mouse secondary antibodies conjugated to alkaline phosphatase (AP) (dilution 1∶10,000; Promega, Madison, WI), and visualized by use of the chromogenic substrates 5-bromo-4-chloro-3′-indolyphosphate (BCIP) and nitro-blue tetrazolium (NBT) (Sigma-Aldrich, St. Louis, MO).

## Results

### Identification of mammalian cell surface receptors for *L. interrogans*

**Figure 1:**
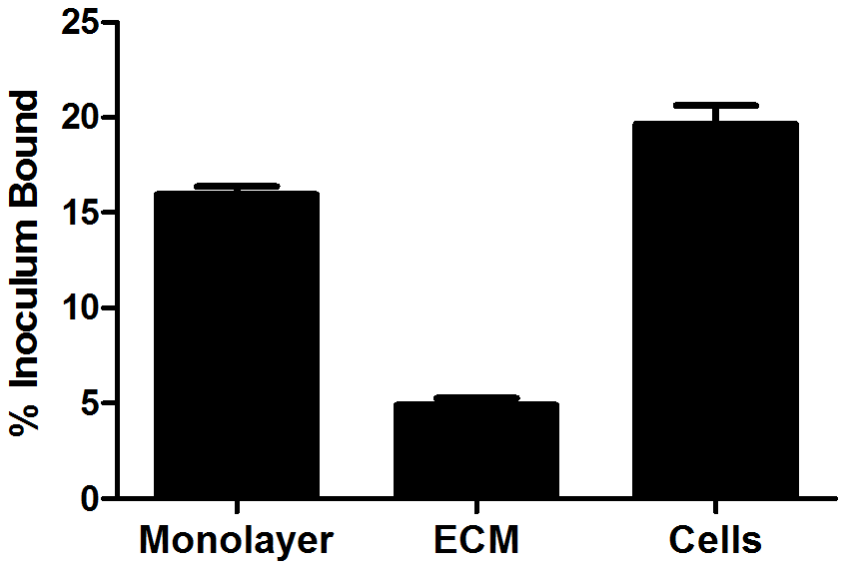
*Leptospira interrogans* binds to EA.hy926 endothelial cells more efficiently than to ECM. Confluent cell layers were left intact, or lifted with EDTA to remove all cells from the ECM without degrading receptors. The cells were pelleted, and both the cells and the ECM were washed in medium without antibiotics prior to the addition of ^35^S-labeled *L. interrogans* serovar Copenhageni strain Fiocruz L1–130 at an MOI of 10. After 1 hr at 37°C followed by washing, bound bacteria were quantified. Results are shown as the mean ± standard error of 112 replicates from multiple experiments. By one way ANOVA followed by Tukey's multiple comparison test, binding to lifted cells vs. the intact layer was not significantly different, but for binding to ECM vs. either lifted cells or intact layer, *P*<0.001.

**Figure 2:**
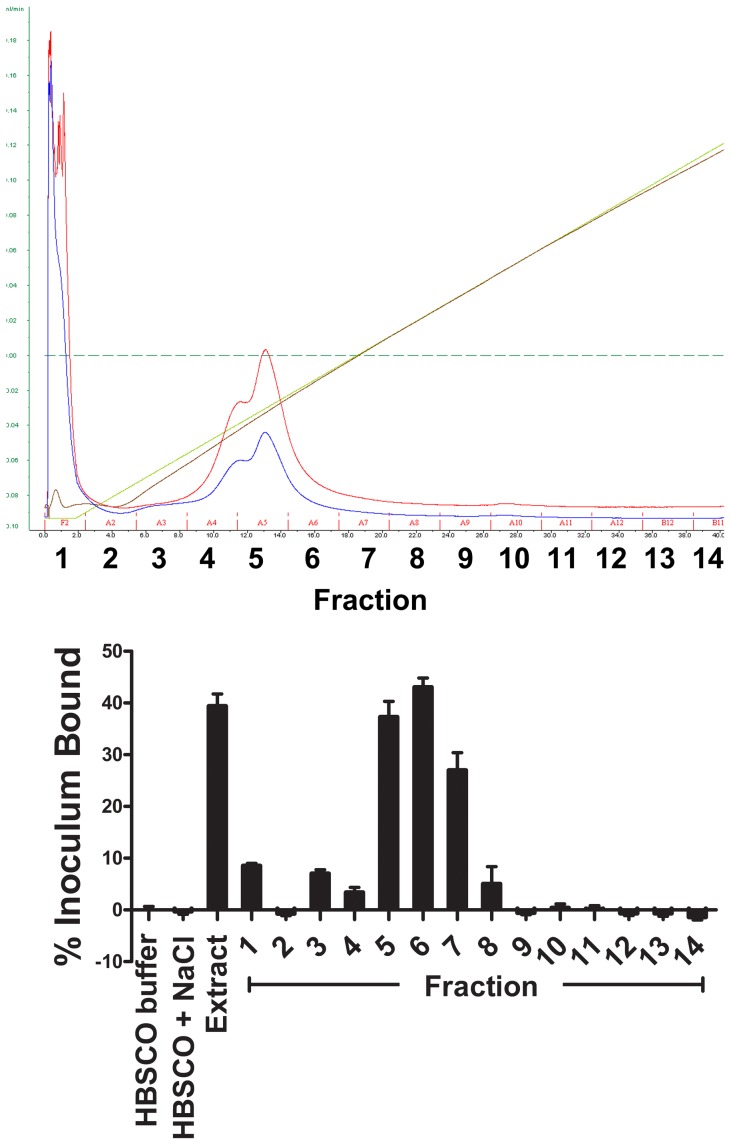
Representative DEAE-sepharose fractionation of EA.hy926 endothelial cell membrane extracts to identify fractions to which *L. interrogans* bound. Top panel: FPLC system traces. The red trace denotes absorbance at 254 nm, the blue trace denotes absorbance at 280 nm, the brown trace denotes conductivity. Bottom panel: Binding of *L. interrogans* to fractions shown in the top panel. The “extract” was the octylglucoside supernatant loaded on to the column. All fractions were diluted 1∶10 in HBSC buffer without detergent prior to dispensing into the wells. After blocking the wells, 3.5×10^5^ ^35^S-labeled pathogenic *L. interrogans* serovar Copenhageni was added to wells, and the plate was incubated 1 hour at 37°C, 5% CO_2_ prior to removal of non-attached bacteria by washing. Buffer with octylglycoside, without or with NaCl, was used as a control for the start and end of the NaCl gradient, respectively.

While a number of laboratories have reported that leptospires bind to cell layers grown *in vitro*, most investigations have focused on interactions of these bacteria with serum or extracellular matrix proteins. To begin to identify mammalian cell surface receptors for *L. interrogans*, we used a macrovascular endothelial cell line, EA.hy926, to which the pathogen *L. interrogans* serovar Copenhageni Fiocruz L1–130 binds efficiently (Figure 1). As previously reported for this and other cell lines [9], binding to the cells is considerably more efficient than to the ECM deposited by the cells. Because our previous work had suggested that *L. interrogans* binds chondroitin sulfate proteoglycans and additional, non-proteoglycan receptors, we set out to identify proteinaceous receptors for *L. interrogans*. Extracts of EA.hy926 endothelial cell membranes were fractionated on a DEAE-sepharose column over a continuous NaCl gradient, and the fractions tested for the ability to bind radiolabeled *L. interrogans* (representative data shown in Figure 2). Fractions with maximal leptospiral binding activity were analyzed by gel electrophoresis followed by LC/MS-based identification of the bands following trypsin digestion. Several bands were unique to the fractions with *L. interrogans* binding activity; we profiled 16 bands that were well-separated in the gels and reproducible between two independent fractionations of EA.hy926 cells. There were additional bands that could not be resolved sufficiently for excision from the gel, regardless of the percentage of acrylamide in the gels, and therefore were not analyzed. LC/MS identifications revealed that the active fractions contained proteins present in adherens junction complexes, i.e. cadherin family members and associated intracellular proteins (Table 1). Other components present in these fractions were a variety of cytokeratins types I and II. These were not considered relevant for further evaluation as part of this work, as these are primarily intracellular intermediate filament proteins.

**Table 1:** Proteins identified by LC/MS in endothelial membrane fractions to which *L. interrogans* bound efficiently.

*Protein*	*number of peptides*	*percent coverage*
desmoplakin* b	17	6.5
junction plakoglobin* b	7	11.2
annexin A2^b^	5	16.8
desmoglein-1* #	5	6.5
annexin A1^b^	4	17.3
actin, cytoplasmic 2 (γ-actin)* a	3	10.1
actin, cytoplasmic 1 (β-actin)* a	3	10.1
plakophilin-1^b^	3	4.4

Data shown are from one of two independent fractionations of EA.hy926 cell extracts on DEAE-sepharose. Keratin and trypsin fragments present in the fractions that bound *L. interrogans* were also found in fractions with no binding activity, and not considered likely to be biologically relevant, so were eliminated from further analysis.

* Proteins identified in both independent experiments.

# Protein is expressed on the surface of intact cells.

a Cytoskeletal protein.

b Adherens junction adaptor protein.

**Figure 3:**
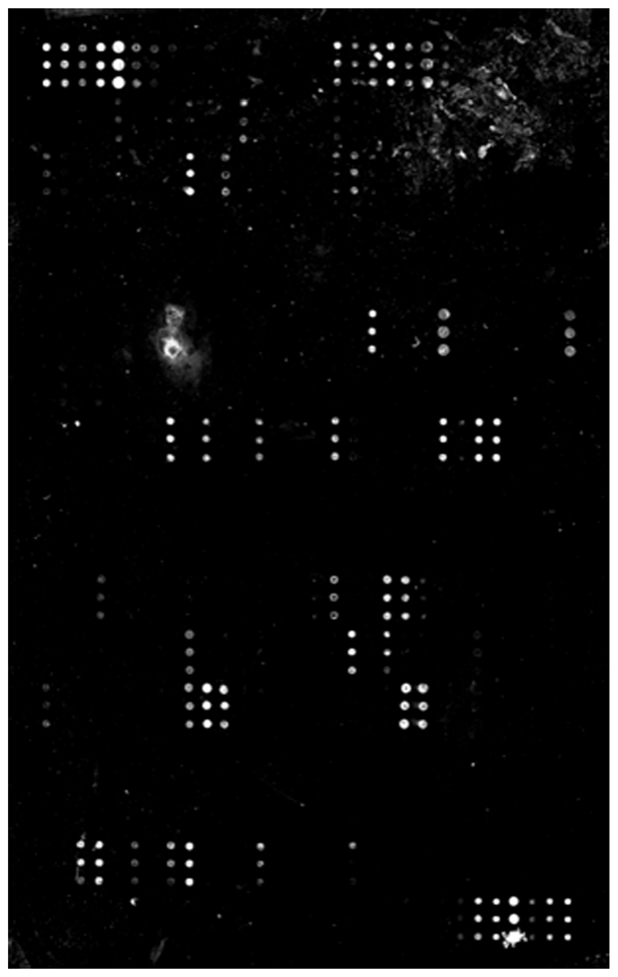
*L. interrogans* serovar Copenhageni Fiocruz L1–130 binds to a specific subset of human proteins in an array. After incubation with the bacteria, the slides were washed and probed with anti-*Leptospira* protein LipL32, then with fluorophore-conjugated detection reagent. Shown is the human protein array probed with *L. interrogans* serovar Copenhageni for 3 hours. The complete data set is presented in Supplemental Table S1.

As a second approach to the identification of human cell surface proteins that could serve as receptors for *L. interrogans*, we employed commercially available protein arrays. The arrays contain 234 different protein targets, as well as both negative and positive controls for the kit-supplied detection reagents. The arrays were probed with *L. interrogans* serovars Copenhageni (strain Fiocruz L1–130) and Canicola (strain 23606) for 1 or 3 hours as per our usual protocol for adhesion studies. The results for the 1 and 3 hour incubations were internally consistent. Fluorescence signals for the 3-hour time point are shown in Figure 3, Table 2 (data are shown after background subtraction), and Table S1, and support cadherins as substrates for *L. interrogans* attachment. Although the mass spectrometry results did not identify VE-cadherin, immunoblot analysis of the EA.hy926 fractions separated by DEAE-sepharose chromatography indicate VE-cadherin was present is some but not all of those with *L. interrogans* binding activity (data not shown). Interestingly, however, the array results also showed high *L. interrogans* attachment to several of the TNF receptor superfamily (TNFRsf) members, and to ICAM-1, -2, and -3. Thus two independent sets of data support the involvement of cadherins in *L. interrogans* attachment to endothelial cells. Additional candidate receptors identified using this approach will be further evaluated in future work.

**Table 2:** Binding of *L. interrogans* to select proteins in the microarray.

Protein	*L. interrogans* sv. Copenhageni	*L. interrogans* sv. Canicola
	(signal-background)	
*TNFR superfamily*		
TROY	13, 737	8,916
TRAIL R2	9,908	7,048
OPG	9,872	4,669
GITR	8,411	4,058
TRAIL R1	5,958	6,762
NGFR	4,207	3,914
HVEM	3,818	4,786
TRAIL R4	2,797	1,976
CD30	2,313	2,498
CD40	1,183	1,404
*Cadherins*		
P-cadherin	4,166	1,731
VE-cadherin	2,797	1,976
E-cadherin	444	146
*ICAMs*		
ICAM-1	1,109	719
ICAM-3	903	1,247
ICAM-2	214	259
*Kit negative control*	1	54
*Kit positive control*	4,726	4,726

Data are shown for the 3 hour time point. The full data set is available online in Table S1.

### Attachment of *Leptospira* to purified cadherins

**Figure 4:**
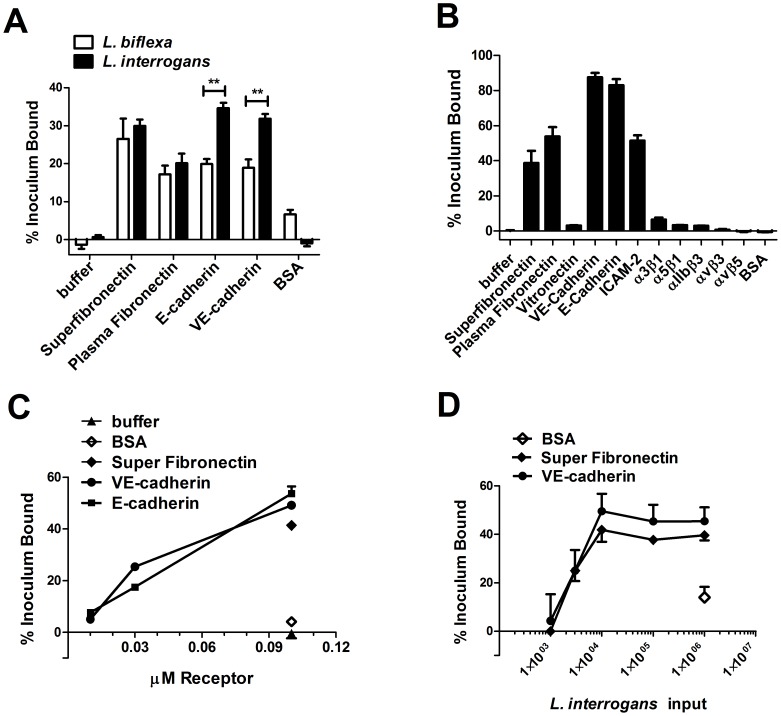
Pathogenic *L. interrogans* binds to purified cadherins. Panel A: Purified receptors were diluted to 0.1 µM in HBSC buffer and immobilized in 96 well plates, and non-specific binding sites were blocked. 3.5×10^5^ ^35^S-labeled pathogenic *L. interrogans* serovar Copenhageni or saprophytic *L. biflexa* serovar Patoc was added to wells, and the plate was incubated 1 hour at 37°C, 5% CO_2_ prior to removal of non-attached bacteria by washing. Bound bacteria were quantified by scintillation counting. Results are shown as the mean ± standard error of 4 replicate determinations of the percent inoculum bound; similar results were obtained from 2 independent experiments. Pathogenic *Leptospira* bind more efficiently to E-cadherin and VE-cadherin compared to the saprophytic strain (Two-way ANOVA, Bonferroni post-test, *P*<0.01). Panel B: The purified proteins shown along the x axis were diluted to 0.1 µM in buffer and immobilized in 96 well plates, and attachment of ^35^S-labeled *L. interrogans* Copenhageni was assessed by methods described for panel A. Binding of *L. interrogans* to each cadherin was significantly more efficient than to fibronectin, the positive control (Tukey's multiple comparison test, P<0.05). Panel C: Purified VE-cadherin and E-cadherin were diluted to 0.01, 0.03 and 0.1 µM in HBSC, immobilized in 96-well plates, and then incubated with 3.5×10^5^ radiolabeled bacteria for 1 hr at 37°C, 5% CO_2_. A dose-dependent attachment of ^35^S *L. interrogans* was observed with increasing concentrations of receptors on the wells. Panel D: Increasing numbers of bacteria (1×10^3^–1×10^6^) were incubated with 0.03 µM VE-cadherin or control super fibronectin immobilized on 96 well plates. The bacterial attachment to VE-cadherin was saturable at 1×10^4^ leptospires. The percent inoculum bound for Panels C and D was determined as previously described in Panel A and results are shown as the mean ± standard error of 4 replicates. For all panels, bovine serum albumin (BSA) served as negative control.

To directly test the possibility that cadherins are receptors for *L. interrogans*, we tested two purified, commercially available cadherins, VE- and E-cadherin, for *L. interrogans* binding by immobilizing the proteins in 96 well plastic dishes and probing with ^35^S-labeled *L. interrogans*. In parallel, we also tested the non-pathogenic *L. biflexa* serovar Patoc, which binds less efficiently to endothelial cells than does *L. interrogans* [9], [11]. *L. interrogans* bound significantly more efficiently than *L. biflexa* to both VE- and E- cadherins (Figure 4A), while there was no significant difference between *L. interrogans* and *L. biflexa* attachment to fibronectin.

A number of additional purified cell-surface proteins were also tested for binding by *L. interrogans*, including integrins α_IIb_β_3_, α_v_β_3_, α_3_β_1_, and α_5_β_1_ (to which *B. burgdorferi* binds [39], [47]–[49]), and BSA as a negative control. As shown in Figure 4B, *L. interrogans* bound efficiently to both cadherins tested. We also observed a direct relationship between bacterial attachment and the amount of purified cadherin added to the plate (Figure 4C). Finally, as depicted in Figure 4D, increasing numbers of *L. interrogans* were incubated with a constant amount of VE-cadherin. Maximal bacterial adherence was observed when 1×10^4^ leptospires were incubated with 0.03 µM receptor. The dose-dependent and saturable binding of *L. interrogans* to VE-cadherin indicates that the receptor-bacteria interaction is specific. In addition, *L. interrogans* bound efficiently to ICAM-2, and to fibronectin, which was used as a positive control. Attachment to the integrins was negligible, further demonstrating that *L. interrogans*-cadherin interactions are specific.

### Expression of cadherins by cell monolayers that bind *L. interrogans*

**Figure 5:**
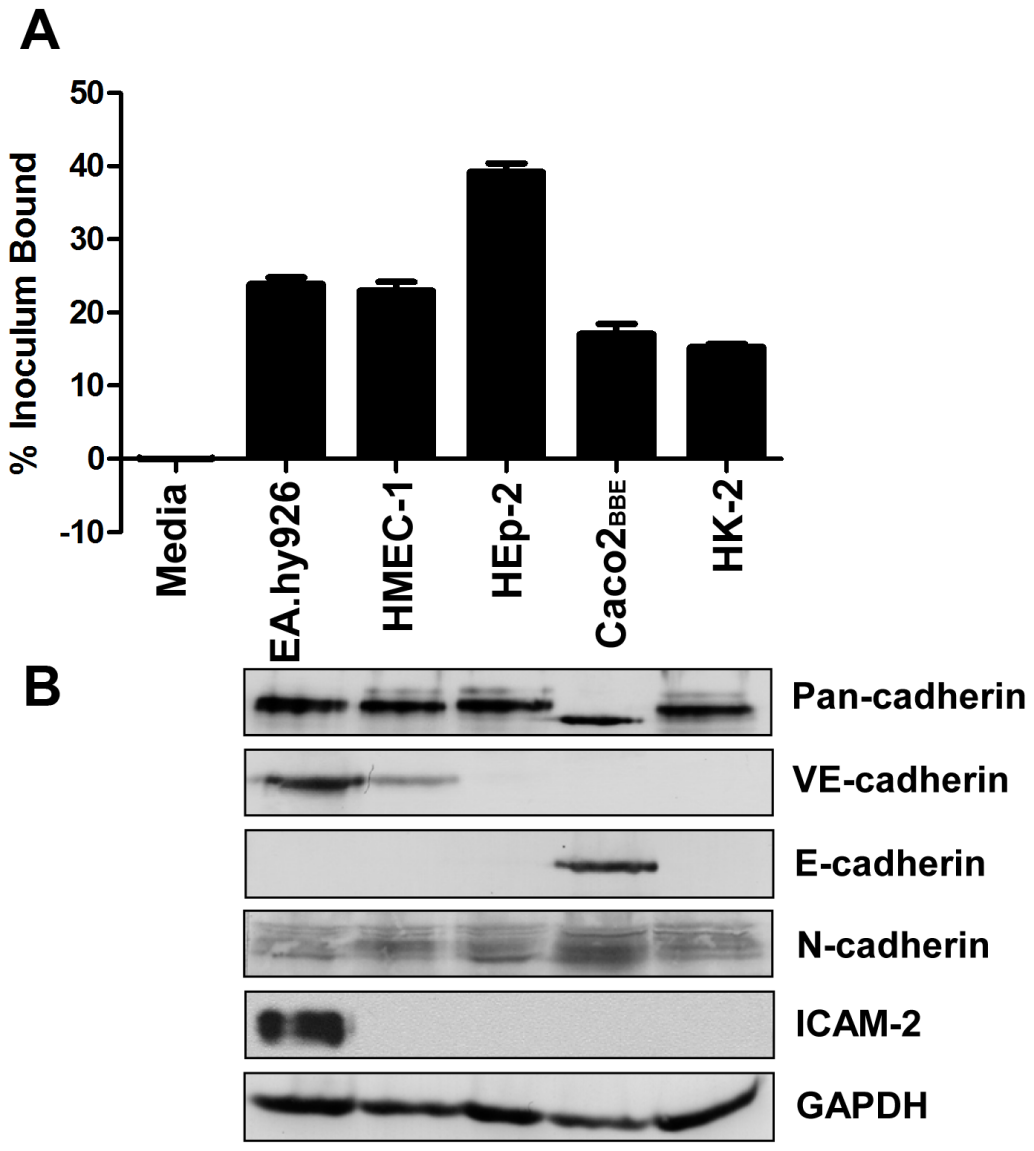
*L. interrogans* binds to cell monolayers expressing cadherins. Panel A: Post-confluent endothelial and epithelial monolayers were incubated with ^35^S labeled *L. interrogans* serovar Copenhageni strain Fiocruz L1–130 at an MOI of 20. After 1 hr at 37°C followed by washing, bacteria bound to monolayers were quantified and expressed as the mean ± standard error of 8–24 replicates from multiple independent experiments. Panel B: Whole cell lysates were prepared from post-confluent cell monolayers. The cells were collected by scraping followed by treatment with lysis buffer. Fifteen micrograms of the lysate were separated by 10% SDS-PAGE and transferred to an Immobilon membrane. The membranes were probed with antibodies against pan-cadherin (dilution 1∶250), VE-cadherin (dilution 1∶100), E-cadherin (dilution 1∶500), N-cadherin (dilution 1∶200), and ICAM-2 (dilution 1∶2,000). A replicate membrane was probed with anti-GAPDH antibody (dilution 1∶1,000) as a loading control. Molecular sizes of bands were determined by comparing their relative mobilities against the set of standards. Band sizes correspond with expected sizes described by antibody manufacturers.

The expression of cadherins in cell monolayers that efficiently bind *Leptospira* was assessed by immunoblot analysis. We surveyed a panel of epithelial and endothelial monolayers, as the ability of pathogenic *Leptospira* to bind to EA.hy926 (human macrovascular endothelial), HMEC-1 (human microvascular endothelial), and HEp-2 (human laryngeal epithelial) monolayers in culture was previously demonstrated [9]. Here, we also show that the human intestinal epithelial cell line Caco2_BBE_ and the human renal proximal tubule cell line HK-2 are able to bind the pathogen (Figure 5A).

We examined the production of receptors by the different cell lines that efficiently bound *L. interrogans.* In Figure 5B, we demonstrated the expression of cadherins in all cell lines using an antibody that detects endogenous levels of cadherins (pan-cadherin; recognizes VE-cadherin, E-cadherin, N-cadherin, P-cadherin, R-cadherin and T-cadherin). The multiple bands observed in HMEC-1, HEp-2 and HK-2 cells suggest the presence of more than one species of cadherin in these cell lines. The band observed in Caco2_BBE_ cell lysate was smaller in size compared to the bands observed from the rest of the cell lines. To determine the expression of specific cadherin receptors, we probed the blots with antibodies against E-cadherin, VE-cadherin and N-cadherin. VE-cadherin expression was observed in both endothelial cell lines examined, but receptor production was more robust in EA.hy926 than HMEC-1 at post-confluence. Among the three epithelial cell lines observed to bind *L. interrogans*, Caco2_BBE_ was the only one in which we were able to detect E-cadherin production. The ∼120 kDa band observed migrated at the same size as the band observed when probed with anti-pan cadherin. N-cadherin, which is widely expressed in endothelial and epithelial cells, was detected in all the cell lines probed. We also examined at the expression of ICAM-2, as both the microarray data and the binding assay result indicate *L. interrogans* binds to this cell surface receptor. Of all cell lines tested, only the macrovascular endothelial cell line EA.hy926 produced ICAM-2.

### Anti-VE-cadherin inhibits attachment of *L. interrogans*

**Figure 6:**
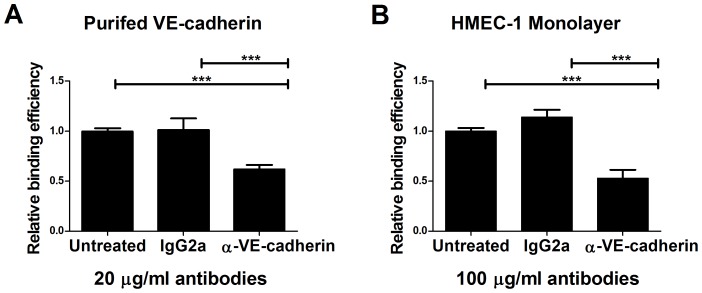
Anti-VE-cadherin inhibits *L. interrogans* attachment to the receptor. Purified VE-cadherin coated on a Linbro plate at 0.1 µM (Panel A) or a post-confluent endothelial cell HMEC-1 monolayer (Panel B) was pretreated with function-blocking anti-VE-cadherin or control antibody IgG2a prior to the addition of 3.5×10^5^ ^35^S-labeled *L. interrogans* Copenhageni Fiocruz L1–130. After 1 hour incubation with *L. interrogans* at 37°C, 5% CO_2_, the unbound bacteria were removed by washing while the bound bacteria were quantified by scintillation counting. The results are expressed as bacterial attachment relative to untreated wells (without antibody) which was set at 1.0, and are shown as mean ± standard error of 8–24 replicates from multiple independent experiments. A significant decrease in the attachment of *L. interrogans* was observed when purified receptor was blocked with 20 µg/ml anti-VE-cadherin and when the HMEC-1 monolayer was pretreated with 100 µg/ml anti-VE-cadherin, but no significant differences were seen with the control IgG2a (Tukey's multiple comparison test, *P*<0.001).

To further assess the interaction of *L. interrogans* with VE-cadherin, we determined whether blocking the receptor using antibodies can inhibit bacterial attachment. Purified receptor diluted to 0.1 µM and immobilized on 96-well plates was pre-treated with increasing concentrations of a function-blocking antibody against VE-cadherin prior to the addition of radiolabeled *L. interrogans.* Inhibition of bacterial attachment was observed when purified receptor was pre-treated with 20 µg/ml anti-VE-cadherin antibody, resulting in a 40% decrease in *L. interrogans* binding (Figure 6A). Pre-treatment of the receptor with an isotype matched (IgG2a) control antibody at the same concentration had no significant effect on *L. interrogans* attachment.

The inhibitory effect of anti-VE-cadherin antibody was also tested on endothelial cell monolayers EA.hy926 and HMEC-1, both of which express the VE-cadherin receptor. In several experiments, the function-blocking antibody against VE-cadherin did not significantly inhibit *L. interrogans* attachment to intact EA.hy926 layers at 100 µg/ml. It should be noted that the production of VE- cadherin was more robust in this cell line compared to HMEC-1 cells, and that ICAM-2 is also produced by EA.hy926 cells. The higher level of VE-cadherin present in EA.hy926 cells, in addition to other *L. interrogans*-receptor interactions (e.g. with ICAM-2 or proteoglycans) may have minimized the effects of the single antibody. However, pre-treatment of post-confluent HMEC-1 monolayers, which do not produce ICAM-2, for 30 minutes with 100 µg/ml anti-VE-cadherin antibody resulted in a 50% reduction in *L. interrogans* binding (Figure 6B), which was not observed when cells were incubated with the IgG2a control antibody.

## Discussion

For many pathogens, including *L. interrogans*, adherence to host cell surfaces is thought of as an initial and important step to establish infection. A number of laboratories have previously published that leptospires bind to cell layers grown *in vitro*, and that, in general, the pathogenic strains bind more efficiently than do non-pathogenic strains [3], [4], [7], [9], [11], [50], [51]. These experiments, however, did not evaluate binding to the cells vs. to the extracellular matrix (ECM) deposited by the cells. We previously established a system to examine *Leptospira*-host cell vs. ECM interactions, specifically [9]. For the work reported here, we used a macrovascular endothelial cell line, EA.hy926 and a microvascular endothelial cell line, HMEC-1, to which the pathogen *L. interrogans* serovar Copenhageni Fiocruz L1–130 binds efficiently. As previously reported [9], binding to the cells is considerably more efficient than to ECM deposited by the cells. Using two independent screening approaches, mass spectrometry and protein array, we identified members of the cadherin family as possible surface receptors for *L. interrogans.* Although the mass spectrometry results did not directly identify VE-cadherin, immunoblot analysis indicates that VE-cadherin is present in some but not all fractions with binding activity (data not shown). Previous work demonstrating that *L. interrogans* binds to proteoglycans [9], as well as the protein array results presented here suggesting that members of the ICAM and TNFR families are potential mammalian host cell surface receptors, support the involvement of multiple pathways mediating *L. interrogans* interactions with mammalian cells. We then focused on the cadherins, and went on to demonstrate that pathogenic *L. interrogans* binds to cadherins significantly more efficiently than does *L. biflexa.* We also showed that the binding of *L. interrogans* to VE-cadherin is dose-dependent and saturable, and that the adherence to purified VE-cadherin and to endothelial cells is significantly inhibited by a function-blocking antibody.

Our finding that cadherins, and possibly ICAMs, serve as receptors for pathogenic *Leptospira* is of interest as other bacterial pathogens have exploited cell adhesion molecules (CAMs) to establish tight contact with the host cells [52], [53]. CAMs are cell surface receptors that mediate cell-cell or cell-ECM attachment and include cadherins, integrins, the immunoglobulin superfamily of CAMs (IgCAMs) and selectins [54]. Cadherins are a family of calcium-dependent transmembrane adhesion proteins [55]. Most cadherins are made up of an extracellular domain that allows cell-cell interaction typically by homophilic adhesion, and a cytoplasmic tail that associates with catenins, forming a link to the cytoskeleton [56]. The ability of cadherins to hold on to receptors of another cell while being anchored to cytoplasmic proteins reflects their fundamental roles in the organization of the intercellular junctions and maintenance of cell-cell cohesion. A prominent example of a bacterial pathogen engaging cadherins is *Listeria monocytogenes.* This Gram-positive, intracellular pathogen expresses internalin (InlA), which binds E-cadherin and mediates adhesion of the bacteria to, and subsequent bacterial internalization by, epithelial cells [51], [57], [58]. Recently, Fardini and colleagues [59] identified VE-cadherin as the receptor for FadA, an adhesin in the oral commensal *Fusobacterium nucleatum.*

We focused our investigation on the role of cadherins, specifically VE-cadherin and E-cadherin, as these receptors are widely expressed on endothelial and epithelial cells, respectively. Both endothelial and epithelial cells represent cell types that are relevant to *Leptospira* infection. As shown by western blot, all cell lines express cadherins when probed with anti-pan cadherin antibody that recognizes the cytoplasmic tails of the receptors. Both EA.hy926 and HMEC-1 express VE-cadherin while Caco2_BBE_ is the only epithelial cell line we examined that produces E-cadherin. All cell lines tested bind *L. interrogans* efficiently, which suggest that in HEp-2 and HK-2 cells, other species of cadherin or distinct cell surface receptors are involved in engaging the pathogen.

Direct interaction of pathogenic *Leptospira* with purified VE-cadherin and E-cadherin receptors was observed *in vitro*. While both the VE-cadherin we purchased and the VE- cadherin spotted on the array showed activity as receptors for *L. interrogans*, E-cadherin in the protein array displayed weaker binding than did the protein in the 96-well plate format. The differing results may be due to different specific activities of the protein preparations, but the relevance of these and all our results will need to be verified by further experimentation *in vivo*.

The dose-dependent and saturable binding of *L. interrogans* to VE-cadherin demonstrate that the interaction is specific. We also demonstrated specificity of the interaction between *L. interrogans* and VE-cadherin through inhibition of bacterial attachment to both the purified receptor and an endothelial cell line by pretreatment with a function-blocking antibody against VE-cadherin. Although inhibition of *Leptospira* binding to HMEC-1 cells was apparent, that was not the case for EA.hy926 cells. By western blot, we observed that VE-cadherin was more abundant in this cell line compared to HMEC-1, suggesting that more receptor molecules may be available to interact with the bacteria even in the presence of antibody. EA.hy926 also expresses ICAM-2, and we showed interaction between the bacteria and purified ICAM-2 *in vitro.* The contribution of ICAM-2 as *Leptospira* receptor on endothelial cells needs to be further evaluated in future work, but it is of interest because it is a well-known ligand for the leukocyte integrin lymphocyte function-associated antigen-1 (LFA-1) on endothelial surfaces [60]. ICAM-2, along with other adhesion molecules E-selectin and VCAM, has been shown to be highly expressed in lungs of patients that succumbed to leptospirosis [61], and is thought to participate in recruitment of inflammatory cells and release of effectors at the infected site [62].

We were not able to reduce *L. interrogans* binding to Caco2_BBE_ cells with anti-E-cadherin antibodies. This may either be due to the robust expression of the receptor or additional *L. interrogans-*receptor interactions may minimize the effects of a single antibody, consistent with the similar results obtained using the anti-VE-cadherin antibody in EA.hy926 cells. We have observed that *L. interrogans* attachment can be inhibited by glycosaminoglycan (GAG) inhibitor 4-Nitrophenyl β-D-xylopyranoside (β-xyloside) but not by its analog 4-Nitrophenyl α-D-galactopyranoside (α-galactoside) (data not shown), implicating the role of proteoglycans in bacterial binding to Caco2_BBE_ cells. An additional possibility is that the *L. interrogans* ligand(s) for cadherins may bind to the receptors with higher affinity than do the antibodies tested. It is also possible that multivalent interactions allowed by a bacterial ligand with a host receptor result in a very stable attachment.

The mechanisms involved in *Leptospira* pathogenesis are not well elucidated, but it is known that the bacteria have the ability to disseminate within the host during early stages of infection [40]. *L. interrogans* is an extracellular pathogen with no known specialized secretion systems, but a susceptible host may carry heavy bacterial burdens in the bloodstream (at least transiently) and in several organs. It is thought that bacterial dissemination is enhanced by the ability of the bacteria to disrupt endothelial layers. By transwell migration assay, our group previously demonstrated that pathogenic *L. interrogans* but not the saprophytic *L. biflexa* caused disruption of the endothelial monolayer resulting in increased transmigration of bacteria [11]. This corresponds to the widespread endothelial damage with increased vascular permeability observed in leptospirosis patients [63]. As an endothelial junctional protein, VE-cadherin plays a major role in maintaining the barrier properties of the vasculature [64], [65]. It is tempting to speculate that VE-cadherin plays a critical role for the *L. interrogans* to attach to the vasculature, resulting in endothelial damage and facilitating the escape of the pathogen from the bloodstream into different tissues during disseminated infection. We hypothesize that disruption in cell-cell interaction upon *L. interrogans* adherence to endothelium may be due to attachment to VE-cadherin. Bacterial attachment to the receptor may disrupt homophilic receptor interactions, and/or render VE-cadherin more susceptible to enzymatic lysis leading to loss of cell-cell contact. Another possibility is that the interaction of *L. interrogans* with VE-cadherin initiates signaling pathways that ultimately result in loss of endothelial integrity. This maybe the case for *F. nucleatum*, as its binding to human umbilical vein cells (HUVECs) results in translocation of VE-cadherin away from the adherens junction complexes leading to endothelial permeability. This allows the passage of the *F. nucleatum*, and enables other bacterial pathogens such as *E. coli* to cross the endothelium [59].

*L. monocytogenes* attachment to E-cadherin leads to bacterial uptake by the epithelial cells [51], [57], [66]. In contrast, there is no evidence in the literature that *L. interrogans* primarily resides in animal cells during infection. Barocchi and colleagues [12] proposed that in epithelial cells, pathogenic *Leptospira* might be invasive but not intracellular. The bacteria can rapidly translocate through cell layers facilitating rapid dissemination. Whether the initial interaction of the bacteria with host cells through cadherins plays a role in this process needs to be investigated further. However, Barocchi and colleagues [12] did not observe the gross disruption of the cell layers that our group previously observed with endothelial cell layers incubated with pathogenic *Leptospira* [11]. We postulate that the binding of *L. interrogans* to host cells through receptors such as cadherins does not lead to intracellular invasion, but instead, in endothelial cells, activates a signaling cascade that results in cytoskeleton rearrangement or receptor recycling. Both of these events can lead to a loss in endothelial integrity, which corresponds to the endothelial disruption observed *in vitro* and to hemorrhagic events of leptospirosis that occur *in vivo.*

Our finding that *L. interrogans* attaches better to cadherins than does the *L. biflexa* is consistent with previous data showing that pathogenic strains of *Leptospira* bind epithelial and endothelial cells more efficiently than saprophytic strains [2], [5], [6], [9], [11]. Several studies also demonstrated that *L. biflexa* adheres well to inert surfaces such as glass and plastic [8], [10], [67]. Ballard and coworkers [2] suggested that the attachment of *L. biflexa* to hosts is non-specific i.e. that the mechanisms by which the saprophytes bind to inert surfaces contribute to the ability of the bacteria to attach to purified receptors and to cells *in vitro.* In contrast, pathogenic *L. interrogans* attachment to endothelial cells *in vitro* results in the disruption of the monolayer [11]. We propose that *L. interrogans* recognizes specific receptors on the host cells, e.g. VE-cadherin, resulting in a signaling cascade leading to loss of endothelial integrity. This possibility will be further investigated in future work.

The role of N-cadherin as a substrate for *L. interrogans* attachment also needs to be explored, as this receptor is produced by both epithelial and endothelial cells, as well as other cell types. Although N-cadherin is co-expressed with VE-cadherin in the endothelia, VE-cadherin is typically located at the intercellular junctions while N-cadherin is largely distributed across the cell surface [68]. N-cadherin is also the predominant cadherin in the renal proximal tubules but is not expressed in other nephron segments [69]. Cadherins may therefore be relevant to *L. interrogans* attachment to the vasculature and to proximal tubules of the kidney, the site of persistent colonization, *in vivo*.

In summary, the study reported here demonstrates the role of VE-cadherin as a receptor of pathogenic *L. interrogans* in endothelial cells. As a major determinant for maintenance and control of endothelial cell contacts, we hypothesize that VE-cadherin interaction with the pathogen activates signaling pathway leading to loss of vascular integrity, facilitating bacterial dissemination. The specific bacterial adhesin(s) that mediate(s) this interaction warrant(s) identification. Understanding the mammalian receptor-bacterial ligand interactions can lead to development of vaccines targeting bacterial proteins involved during early stages of infection. The cadherins, are the first cell surface proteins identified as receptors for *L. interrogans*. In addition, the ICAMs, TNFRsf members and other proteins identified in the array warrant further investigation to assess their biological significance.

## Supporting Information

Table S1 **Complete data set of the human protein arrays probed with** ***L. interrogans*** **serovar Copenhageni and** ***L. interrogans*** **serovar Canicola.** Shown are the fluorescence signals (after background subtraction) for the 1 and 3-hour time points. The array coordinates are available from RayBiotech (http://www.raybiotech.com/raybio-human-protein-array-1-2.html”).
(DOCX)
