# Supplementary material for: Leptospira interrogans Binds to Cadherins
Source: PLoS Negl Trop Dis. 2014 Jan 30;8(1):e2672. doi: 10.1371/journal.pntd.0002672 (PMC3907533; doi:10.1371/journal.pntd.0002672)
Supplement: Table S1 — Complete data set of the human protein arrays probed with L. interrogans serovar Copenhageni and L. interrogans serovar Canicola. Shown are the fluorescence signals (after background subtraction) for the 1 and 3-hour time points. The array coordinates are available from RayBiotech (http://www.raybiotech.com/raybio-human-protein-array-1-2.html”). (DOCX) [file pntd.0002672.s001.docx]

**Supplementary Table 1. Complete data set of the human protein arrays probed with *L. interrogans* serovar Copenhageni and *L. interrogans* serovar Canicola.** Shown are the fluorescence signals (after background subtraction) for the 1 and 3-hour time points.

|  |  | **Copenhageni** | | **Canicola** | |
| --- | --- | --- | --- | --- | --- |
|  |  | **1 Hr** | **3 Hr** | **1 Hr** | **3 Hr** |
| 1 | **Positive Control** | 4,726 | 4,726 | 4,726 | 4,726 |
| 2 | **Neg** | 39 | 1 | 20 | 54 |
| 3 | **4-IBBL** | 167 | 39 | 19 | 22 |
| 4 | **4-IBBR** | 1 | 1 | 1 | 1 |
| 5 | **Acrp30** | 1 | 5 | 1 | 13 |
| 6 | **Activin A** | 476 | 1 | 42 | 1 |
| 7 | **ALCAM** | 1,296 | 2,213 | 3,458 | 2,855 |
| 8 | **Angiogenin** | 1 | 21 | 473 | 1 |
| 9 | **Angiopoietin-1** | 1 | 170 | 371 | 197 |
| 10 | **Angiopoietin-2** | 42 | 637 | 964 | 2,148 |
| 11 | **Angiostatin** | 1 | 581 | 337 | 820 |
| 12 | **AR (Amphiregulin)** | 66 | 1 | 1 | 1 |
| 13 | **ART** | 1 | 1 | 1 | 95 |
| 14 | **AXL** | 1,509 | 3,778 | 2,040 | 2,290 |
| 15 | **b NGF** | 254 | 156 | 1,523 | 489 |
| 16 | **B7-1 (CD 80)** | 1,238 | 1,501 | 861 | 2,408 |
| 17 | **BCAM** | 16 | 332 | 4 | 312 |
| 18 | **BDNF** | 243 | 1 | 651 | 1 |
| 19 | **BLC** | 22 | 1 | 245 | 1 |
| 20 | **BMP-4** | 323 | 36 | 163 | 108 |
| 21 | **BMP-5** | 303 | 1 | 1 | 1 |
| 22 | **BMP-6** | 22 | 1 | 69 | 1 |
| 23 | **BMP-7** | 71 | 1 | 1 | 92 |
| 24 | **BTC** | 1 | 1 | 1 | 735 |
| 25 | **Cardiotrophin-1 (CT-1)** | 51 | 42 | 66 | 1 |
| 26 | **Cathepsin S** | 1 | 1 | 53 | 1 |
| 27 | **CCL21 / 6Ckine** | 1 | 1 | 1 | 1 |
| 28 | **CD14** | 1 | 1 | 1 | 1 |
| 29 | **CD27** | 1 | 19 | 56 | 720 |
| 30 | **CD30** | 152 | 2,313 | 1,098 | 2,498 |
| 31 | **CD40** | 618 | 1,183 | 1,690 | 1,404 |
| 32 | **CD40 Ligand** | 1 | 16 | 1 | 9 |
| 33 | **CK beta 8-1 / CCL23** | 204 | 1 | 67 | 1 |
| 34 | **CNTF** | 1 | 507 | 18 | 72 |
| 35 | **Complement Factor D** | 1 | 188 | 77 | 52 |
| 36 | **C-peptide** | 1 | 1 | 3 | 1 |
| 37 | **CTACK** | 19 | 132 | 545 | 266 |
| 38 | **CTLA-4** | 8,487 | 15,942 | 9,223 | 11,694 |
| 39 | **CXCL-16** | 6 | 38 | 63 | 7 |
| 40 | **DAN** | 1,146 | 3,349 | 2,559 | 1,986 |
| 41 | **DKK-1** | 1 | 25 | 129 | 340 |
| 42 | **DKK-4** | 142 | 139 | 70 | 518 |
| 43 | **DR 6** | 311 | 1,487 | 47 | 264 |
| 44 | **Dtk** | 1,544 | 4,084 | 987 | 3,114 |
| 45 | **E-Cadherin** | 1 | 444 | 1 | 146 |
| 46 | **EGF** | 1 | 1 | 1 | 43 |
| 47 | **EGF-R** | 15 | 443 | 706 | 1,019 |
| 48 | **ENA-78** | 1 | 1 | 83 | 107 |
| 49 | **Endoglin** | 1 | 1 | 1 | 1 |
| 50 | **Endostatin** | 1 | 1 | 1 | 3 |
| 51 | **Eotaxin** | 545 | 1 | 175 | 1 |
| 52 | **Eotaxin-2** | 1 | 1 | 176 | 71 |
| 53 | **Eotaxin-3** | 82 | 145 | 750 | 166 |
| 54 | **ErbB3** | 1 | 442 | 46 | 365 |
| 55 | **Erythropoietin R** | 1 | 1 | 54 | 1 |
| 56 | **E-selectin** | 599 | 1 | 1 | 99 |
| 57 | **Fas** | 1 | 21 | 140 | 63 |
| 58 | **Fas Ligand** | 1 | 1 | 24 | 2 |
| 59 | **Fc Gamma RII B/C (CD32b/c)** | 99 | 1 | 1 | 52 |
| 60 | **FGF-4** | 1 | 67 | 24 | 7 |
| 61 | **FGF-6** | 12 | 1 | 83 | 10 |
| 62 | **FGF-7** | 32 | 1 | 184 | 122 |
| 63 | **FGF-9** | 1 | 1 | 1 | 319 |
| 64 | **FGF-b** | 585 | 195 | 470 | 1,375 |
| 65 | **FIT-3 Ligand** | 1 | 1 | 1 | 1 |
| 66 | **Follistatin** | 1 | 109 | 54 | 162 |
| 67 | **Fractalkine** | 216 | 1 | 26 | 70 |
| 68 | **GCP-2** | 1 | 61 | 7 | 49 |
| 69 | **GCSF** | 258 | 138 | 478 | 1 |
| 70 | **GDNF** | 99 | 1 | 79 | 1 |
| 71 | **GH** | 1 | 1 | 1 | 42 |
| 72 | **GITR** | 7,376 | 8,411 | 3,563 | 4,058 |
| 73 | **GITR Ligand** | 248 | 332 | 515 | 542 |
| 74 | **GLP-1 (Glucagon Like)** | 232 | 250 | 84 | 1 |
| 75 | **GM-CSF** | 104 | 119 | 1 | 1 |
| 76 | **gp130** | 4,803 | 4,566 | 5,209 | 6,160 |
| 77 | **GRO-a** | 12 | 170 | 1 | 14 |
| 78 | **GRO-b** | 1 | 1 | 1 | 1 |
| 79 | **GRO-r** | 198 | 1 | 1 | 1 |
| 80 | **HB-EGF** | 1 | 17 | 17 | 82 |
| 81 | **HCC-4** | 1 | 1 | 25 | 43 |
| 82 | **HGF** | 1 | 1 | 1 | 1 |
| 83 | **HVEM** | 3,566 | 3,818 | 1,646 | 4,786 |
| 84 | **I-309** | 43 | 17 | 44 | 25 |
| 85 | **ICAM-1** | 931 | 1,109 | 954 | 719 |
| 86 | **ICAM-2** | 501 | 214 | 418 | 259 |
| 87 | **ICAM-3** | 3,362 | 903 | 1,735 | 1,247 |
| 88 | **IFN-gamma** | 120 | 1 | 4 | 3 |
| 89 | **IGF-1R** | 15 | 1 | 1 | 1 |
| 90 | **IGFBP-1** | 82 | 1 | 14 | 1 |
| 91 | **IGFBP-2** | 1 | 1 | 1 | 31 |
| 92 | **IGFBP-3** | 1 | 1 | 1 | 1 |
| 93 | **IGFBP-4** | 1 | 14 | 1 | 1 |
| 94 | **IGFBP-6** | 1 | 1 | 1 | 1 |
| 95 | **IGF-I** | 54 | 1 | 237 | 1 |
| 96 | **IGF-II** | 1 | 1 | 54 | 1 |
| 97 | **IL-1a** | 353 | 398 | 268 | 11 |
| 98 | **IL-1b** | 79 | 1 | 38 | 44 |
| 99 | **IL-1ra** | 1 | 47 | 78 | 45 |
| 100 | **IL-1RI** | 1 | 1 | 1 | 1 |
| 101 | **IL-1RII** | 1 | 1 | 1 | 1 |
| 102 | **IL-2** | 1 | 1 | 1 | 1 |
| 103 | **IL-2R alpha** | 1 | 1 | 1 | 1 |
| 104 | **IL-2R beta** | 4 | 15 | 7 | 1 |
| 105 | **IL-2R gamma** | 1 | 19 | 47 | 15 |
| 106 | **IL-3** | 65 | 14 | 42 | 1 |
| 107 | **IL-4** | 1 | 1 | 1 | 1 |
| 108 | **IL-5** | 1 | 148 | 376 | 447 |
| 109 | **IL-5 R alpha** | 1 | 94 | 102 | 73 |
| 110 | **IL-6** | 390 | 1 | 1 | 1 |
| 111 | **IL-6 sR** | 1 | 1 | 1 | 1 |
| 112 | **IL-7** | 1 | 7 | 97 | 25 |
| 113 | **IL-8** | 1 | 1 | 1 | 1 |
| 114 | **IL-9** | 19 | 7 | 25 | 33 |
| 115 | **IL-9 R** | 1 | 19 | 66 | 127 |
| 116 | **IL-10** | 1 | 1 | 98 | 114 |
| 117 | **IL-10 R alpha** | 588 | 490 | 427 | 427 |
| 118 | **IL-10 R beta** | 11,899 | 8,641 | 8,611 | 5,904 |
| 119 | **IL-11** | 160 | 29 | 1 | 1 |
| 120 | **IL-12p40** | 6,684 | 5,643 | 3,434 | 2,659 |
| 121 | **IL-12p70** | 69 | 14 | 42 | 19 |
| 122 | **IL-13** | 241 | 85 | 408 | 138 |
| 123 | **IL-13 Ra1** | 9,596 | 3,805 | 5,857 | 3,919 |
| 124 | **IL-13 Ra2** | 6,144 | 6,497 | 3,983 | 3,290 |
| 125 | **IL-15** | 688 | 859 | 1,524 | 1,176 |
| 126 | **IL-16** | 1 | 1 | 1 | 1 |
| 127 | **IL-17** | 1 | 9 | 1 | 1 |
| 128 | **IL-17B** | 1 | 1 | 1 | 1 |
| 129 | **IL-17C** | 1 | 1 | 1 | 1 |
| 130 | **IL-18 BPa** | 13,506 | 11,658 | 11,363 | 10,897 |
| 131 | **IL-18 R alpha** | 275 | 785 | 427 | 501 |
| 132 | **IL-18 R beta** | 9,644 | 9,603 | 6,126 | 6,461 |
| 133 | **IL-21R** | 17,134 | 14,909 | 13,192 | 13,382 |
| 134 | **IL-28A / IFN-lambda** | 1 | 1 | 1 | 76 |
| 135 | **Insulin** | 1 | 1 | 1 | 28 |
| 136 | **Insulin-Receptor** | 1 | 48 | 20 | 100 |
| 137 | **IP-10** | 121 | 1 | 1 | 1 |
| 138 | **I-TAC** | 1 | 1 | 1 | 1 |
| 139 | **LAP** | 291 | 128 | 320 | 196 |
| 140 | **Leptin (OB)** | 281 | 159 | 380 | 387 |
| 141 | **Leptin R** | 3,714 | 2,436 | 1,735 | 1,764 |
| 142 | **LIF** | 1 | 34 | 1 | 151 |
| 143 | **Light** | 339 | 112 | 33 | 45 |
| 144 | **L-selectin** | 497 | 33 | 11 | 31 |
| 145 | **Lymphotactin** | 277 | 97 | 48 | 9 |
| 146 | **MCP-1** | 248 | 56 | 66 | 62 |
| 147 | **MCP-2** | 1 | 34 | 1 | 39 |
| 148 | **MCP-3** | 56 | 22 | 13 | 22 |
| 149 | **MCP-4** | 14 | 1 | 1 | 17 |
| 150 | **M-CSF** | 89 | 73 | 1 | 15 |
| 151 | **M-CSF R** | 2,153 | 2,760 | 2,922 | 1,574 |
| 152 | **MDC** | 49 | 90 | 135 | 40 |
| 153 | **MEC (CCL-28 / VIC)** | 226 | 209 | 129 | 49 |
| 154 | **MICA** | 5,737 | 7,565 | 4,789 | 3,369 |
| 155 | **MICB** | 5,687 | 6,721 | 2,016 | 4,782 |
| 156 | **MIF** | 1 | 896 | 129 | 293 |
| 157 | **MIG** | 20 | 103 | 1 | 1 |
| 158 | **MIP-1a** | 216 | 1 | 81 | 83 |
| 159 | **MIP-1b** | 1 | 48 | 1 | 56 |
| 160 | **MIP-1d** | 1 | 1 | 71 | 3 |
| 161 | **MIP-3a** | 16 | 12 | 1 | 32 |
| 162 | **MIP-3b** | 1 | 1 | 310 | 38 |
| 163 | **MMP-1** | 115 | 390 | 383 | 367 |
| 164 | **MMP-2** | 172 | 14 | 383 | 20 |
| 165 | **MMP-3** | 169 | 110 | 14 | 243 |
| 166 | **MMP-8** | 19 | 1 | 55 | 23 |
| 167 | **MMP-9** | 173 | 49 | 86 | 61 |
| 168 | **MMP-10** | 955 | 178 | 274 | 181 |
| 169 | **MMP-13** | 612 | 81 | 166 | 202 |
| 170 | **MPIF-1** | 80 | 1 | 1 | 29 |
| 171 | **MSP** | 1 | 17 | 1 | 99 |
| 172 | **NAP-2** | 12 | 1 | 1 | 89 |
| 173 | **NGF R** | 5,441 | 4,207 | 3,590 | 3,914 |
| 174 | **NRG-I-b1** | 1 | 1 | 32 | 1 |
| 175 | **NT-3** | 35 | 95 | 12 | 182 |
| 176 | **NT-4** | 77 | 93 | 1 | 117 |
| 177 | **OBS (Obstatin-C-terminus)** | 572 | 399 | 379 | 144 |
| 178 | **Oncostatin M (OSM)** | 231 | 397 | 117 | 321 |
| 179 | **Osteoprotegerin (OPG)** | 9,769 | 9,872 | 8,551 | 4,669 |
| 180 | **PARC** | 52 | 214 | 57 | 201 |
| 181 | **P-Cadherin** | 5,745 | 4,166 | 2,368 | 1,731 |
| 182 | **PDGF AA** | 103 | 250 | 1 | 93 |
| 183 | **PDGF AB** | 35 | 307 | 1 | 42 |
| 184 | **PDGF BB** | 1 | 42 | 21 | 12 |
| 185 | **PDGF R alpha** | 1 | 166 | 1 | 205 |
| 186 | **PDGF R beta** | 1,008 | 875 | 706 | 704 |
| 187 | **PECAM-1 (CD31)** | 64 | 1 | 1 | 62 |
| 188 | **PF-4** | 1 | 27 | 1 | 42 |
| 189 | **PIGF** | 73 | 47 | 172 | 63 |
| 190 | **Prolactin** | 1 | 1 | 93 | 59 |
| 191 | **P-selectin** | 93 | 1 | 146 | 163 |
| 192 | **RAGE** | 6,497 | 2,058 | 2,718 | 2,413 |
| 193 | **Rantes** | 322 | 363 | 35 | 56 |
| 194 | **SAA** | 274 | 44 | 274 | 139 |
| 195 | **SCF** | 1 | 1 | 8 | 53 |
| 196 | **SCF R** | 128 | 10 | 48 | 64 |
| 197 | **SDF-1a** | 73 | 1 | 14 | 1 |
| 198 | **SDF-1b** | 1 | 1 | 1 | 12 |
| 199 | **Shh-N** | 61 | 1 | 1 | 48 |
| 200 | **Siglec-5** | 7,155 | 5,457 | 4,445 | 3,804 |
| 201 | **Siglec-9** | 19,295 | 12,366 | 7,690 | 10,389 |
| 202 | **ST2 (IL-1R4)** | 11,668 | 8,106 | 4,292 | 3,995 |
| 203 | **TARC** | 1 | 1 | 44 | 56 |
| 204 | **TECK** | 46 | 107 | 155 | 9 |
| 205 | **TGF-a** | 414 | 155 | 321 | 540 |
| 206 | **TGF-beta 1** | 562 | 157 | 27 | 136 |
| 207 | **TGF-beta 2** | 95 | 1 | 198 | 1 |
| 208 | **TGF-beta 3** | 1 | 95 | 65 | 8 |
| 209 | **Tie-1** | 9,015 | 8,637 | 7,236 | 4,314 |
| 210 | **Tie-2** | 7,026 | 6,589 | 5,321 | 5,028 |
| 211 | **Timp-1** | 22 | 36 | 5 | 31 |
| 212 | **Timp-2** | 66 | 1 | 1 | 1 |
| 213 | **Timp-3** | 183 | 233 | 364 | 476 |
| 214 | **Timp-4** | 1 | 3 | 41 | 1 |
| 215 | **TNF-a** | 1 | 119 | 10 | 1 |
| 216 | **TNF-b** | 49 | 76 | 74 | 73 |
| 217 | **TNF-R I** | 46 | 1 | 10 | 1 |
| 218 | **TNF-R II** | 1 | 48 | 15 | 1 |
| 219 | **TPO** | 146 | 1 | 1 | 223 |
| 220 | **TRAIL** | 23 | 71 | 1 | 160 |
| 221 | **TRAIL R1** | 18,008 | 5,958 | 8,656 | 6,762 |
| 222 | **TRAIL R2** | 20,885 | 9,908 | 11,535 | 7,048 |
| 223 | **TRAIL R3** | 1 | 23 | 209 | 178 |
| 224 | **TRAIL R4** | 6,506 | 2,797 | 3,837 | 1,976 |
| 225 | **TRANCE** | 144 | 1 | 1 | 1 |
| 226 | **TREM-1** | 5,303 | 4,005 | 3,233 | 1,396 |
| 227 | **TROY** | 14,356 | 13,737 | 12,979 | 8,916 |
| 228 | **u PAR** | 1 | 1 | 1 | 1 |
| 229 | **ubiquitin+1** | 123 | 49 | 189 | 68 |
| 230 | **VCAM-1** | 1,049 | 66 | 1 | 92 |
| 231 | **VE- Cadherin** | 4,743 | 3,556 | 1,735 | 467 |
| 232 | **VEGF** | 39 | 25 | 14 | 64 |
| 233 | **VEGF R2** | 1,876 | 939 | 687 | 594 |
| 234 | **VEGF R3** | 77 | 31 | 1 | 1 |
| 235 | **VEGF-D** | 127 | 62 | 450 | 211 |
| 236 | **VEGI** | 1 | 35 | 18 | 1 |
